# Diophantine imaging reveals the broken symmetry of sums of integer cubes

**DOI:** 10.1038/s41598-023-49960-y

**Published:** 2024-01-02

**Authors:** Eldar Sultanow, Max Henkel, Idriss J. Aberkane

**Affiliations:** 1 Capgemini, Bahnhofstraße 30, 90402 Nuremberg, Germany; 2 Schmalkalden University of Applied Sciences, Blechhammer 9, 98574 Schmalkalden, Germany; 3 Scanderia online education, 66 av. des Champs-Elysées, 75008 Paris, France; 4 Bioniria Foundation, Place Numa-Droz 2, 2000 Neuchâtel, Switzerland; 5 UNESCO-UniTwin Complex Systems Digital Campus, ECCE e-Lab, Strasbourg Université CEDEX, 67081 Strasbourg, France

**Keywords:** Engineering, Mathematics and computing

## Abstract

We introduced a novel method for visualizing large diophantine datasets and in particular found that mapping the known integer triplets $\{a,b,c\}$ solving either equations of the type $a^3+b^3+c^3=d$ or $a^3+b^3+c^3=d^3$ on certain proper subgroups of the circle group exposed a very clear breaking in their symmetry and a strongly non-ergodic distribution of the solutions of sums of three cubes that had never been described before. This method could be further applied to a larger diversity of diophantine problems, informing both number-theoretical conjectures and novel methods in computer sciences on the way, along with paving the road for specific uses of machine learning in exploring diophantine datasets with possible applications in cryptography among others.

## Introduction

Diophantine equations are used in cryptosystems, since they play a significant role in cryptography, particularly in the realm of public-key cryptography^1^^[,2,3^. The complexity and computational difficulty of solving Diophantine equations, make them ideal for creating cryptographic keys that are easy to generate but extremely hard to reverse-engineer. The larger the search space, the harder it is to retrieve a solution. This inherent difficulty provides a secure foundation for encrypting data and secure communications.

For an integer *d*, finding the three integers $\{a,b,c\}$ that verify $a^3+b^3+c^3=d$ is both a very stubborn problem of contemporary mathematics and computational science; the first ever solution for number 33 was only found in 2019 for example^4^. The so-called cubic quadruples $(a^3+b^3+c^3=d^3)$ form another class of diophantine problems which, though non-trivial, are considered significantly simpler^5,]^^6^. Offering new visual insight into difficult diophantine equations such as the sums of integer cubes could lead to new advances in Number theory and significantly improve the efficiency of certain search algorithms by narrowing their search space. It is the purpose of this article to introduce such a form of geometric representation - which we call “diophantine imaging” along with what we consider one of its most significant low-hanging algebraic fruits, that is, evidence for the strongly negentropic distribution of the solutions of sums of integer cubes respective to that of cubic quadruples.

Commenting on the methodology of the Green-Tao theorem, Matheus^7^ has called ergodic theory “remarkably effective” in Number theory, which remains an understatement. The purpose of this paper is to introduce the use of a certain coordinate system within the circle group and some of its discrete subgroups (like the Prüfer 2-group) to study the known solutions of two important diophantine problems: the sums of three cubes and cubic quadruples, and investigate some essential differences in their distributions, especially respective to their prefixes in base *p*. In particular, we show that while the solutions of sums of three cubes are distributed in a negentropic manner (Here we will simply understand “negentropic” as “significantly deviating from a random distribution), which observation and characterization could constitute a further research program, the comparative entropy in the distribution of cubic quadruples leads us to conjecture they could be organized as conference graphs.

## Methods

### Origin and purpose

We call “Diophantine Imaging” a method of arithmetic geometry by which solutions of diophantine equations are represented over the unit circle based on their prefixes in certain bases. The purpose of this method is to keep the representation contained for arbitrarily large numbers and maintain an intuitive metric based on converging series of angular distances, which in itself is borrowed from p-adic arithmetic. Since we intend to make this method accessible to the widest possible audience in both mathematics and computer science, we will be adding here even the most basic textbook references as we walk the reader through, assuming they do not necessarily have a background in p-adic arithmetic, Prüfer groups and the like.

**Figure 1:**
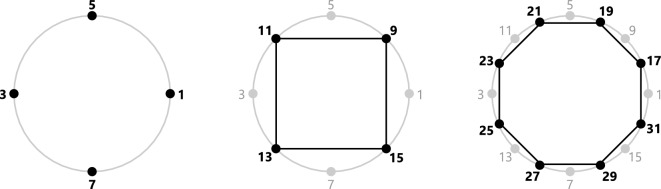
Counterclockwise labeling of distinguished vertices within the unit circle. Shown here is the base 2 case, which is isomorphic to a Prüfer 2-group.

Let us begin by taking the unit circle in ${\mathbb {C}}$ and labelling distinguished n-th roots of unity with odd natural numbers, counterclockwise as shown in Figure 1. The identity element 1 is located at angle $2\pi$, opposite to it, at angle $\pi$, is number 3 for the base 2 case. To place and label all odd integers on the unit circle, we then embed successive regular *n*-gons in it, each covering numbers from $2^{n+1}+1$ to $2^{n+2}-1$. For the integers 9 to 15 the n-gon is a square, for 17 to 31 it is an octagon, for 33 to 63 a hexadecagon, and so forth.

**Figure 2:**
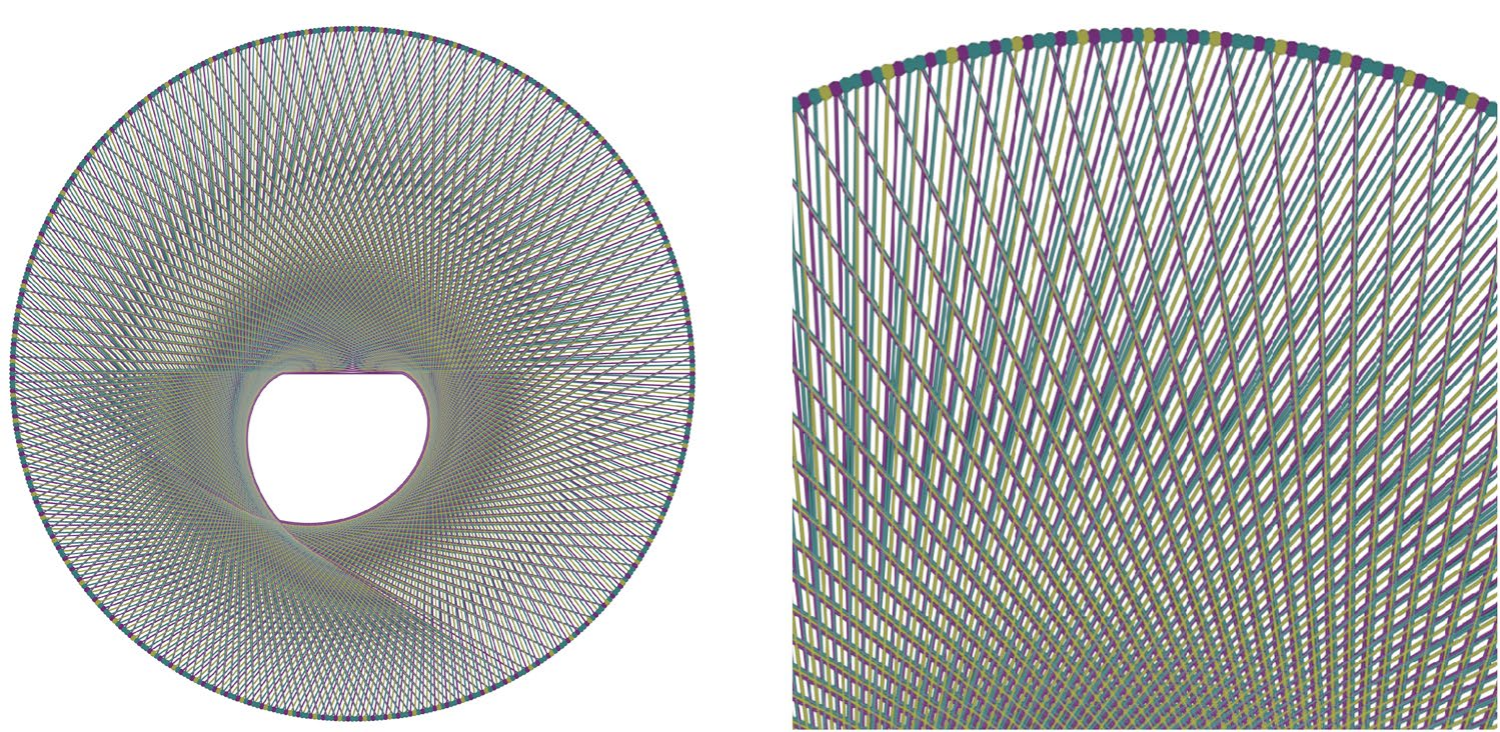
The *dreamcatcher of action* $\times 3$ *in base 2* is the envelope generated by connecting each element of our labeled Prüfer 2-group to its multiplication by 3. Note here that each element is colored according to its residue class in base 3: elements in $[2]_3$ are shown in teal, those in $[1]_3$ in purple and those in $[0]_3$ in yellow^8^ A zoom is shown on the right to better illustrate the color coding.

Originally, this coordinate system was introduced by the last author to study both the Collatz and Furstenberg $\times 2 \times 3$ conjecture; in particular, its purpose was to generate the specific envelope associated with operation $\times 3$ over the base 2 representation of a given number. The last author called this novel variety of envelopes “dreamcatchers” both in reference to the eponymous Anishinaabe first nation talismans and to Kronecker’s *liebster Jugendtraum* and we already demonstrated in^8^ some of its interests in arithmetic geometry. For example, in the same referential, the dreamcatcher of action $\times 3$ in base 2 is depicted by Figure 2.

### Algebraic formalism

Let us now exhaustively describe the method of diophantine imaging for both the working computer scientist and number theorist, and in particular deduce the group operator in the base 2 case, that is, in our labeled Prüfer 2-group. An illustrative and formal definition of the Prüfer 2-Group is already given by John Baez^9^, to which we will refer below. Most simply put, the Prüfer 2-Group is the set of cosets of rationals whose denominator is a power of 2: $${\mathbb {Z}}(2^\infty )={\mathbb {Z}}[ {\raise0.7ex\hbox{$1$} \!\mathord{\left/ {\vphantom {1 2}}\right.\kern-\nulldelimiterspace} \!\lower0.7ex\hbox{$2$}} ]/{\mathbb {Z}}$$, see^10^, p. 16^11^, p. 8. Comfort, Hofmann and Remus^12^ also describe the Prüfer *p*-Group as the union $$\cup _{n\in {\mathbb {N}}}{\mathbb {Z}}(p^n)$$, where $${\mathbb {Z}}(n)$$ denotes the cyclic group of order *n* (which acts as a plane rotation group on the set of *n* vertices of a regular *n*-gon^13^, p. 203^14^, p. 58). The *n*-gons are in the context of the Prüfer *p*-Group $$p^n$$-gons as plotted in Figure 1 for the particular case $$p=2$$.

Baez denotes the elements of the Prüfer 2-Group by $$g_1,g_2,\ldots$$ and the identity by *e*. The elements satisfy the relations $$g_1^2=1$$, $$g_2^2=g_1$$, $$g_3^2=g_2$$ and so forth. To illustrate the Prüfer 2-Group geometrically, the group elements are located on the unit circle of complex numbers: $$e=1$$, $$g_1=e^{i\pi }$$, $$g_2=e^{ {\raise0.7ex\hbox{${i\pi }$} \!\mathord{\left/ {\vphantom {{i\pi } 2}}\right.\kern-\nulldelimiterspace} \!\lower0.7ex\hbox{$2$}} }$$, $$g_3=e^{ {\raise0.7ex\hbox{${i\pi }$} \!\mathord{\left/ {\vphantom {{i\pi } 4}}\right.\kern-\nulldelimiterspace} \!\lower0.7ex\hbox{$4$}} }$$, and so on. Now let’s uniquely assign our labels to the group elements. The identity element is $$e=1$$ and its angle in the unit circle is $$0\pi$$. The element $$g_1=3$$, its angle in the unit circle is $$1\pi$$. The coset $${\raise0.7ex\hbox{$1$} \!\mathord{\left/ {\vphantom {1 2}}\right.\kern-\nulldelimiterspace} \!\lower0.7ex\hbox{$2$}}+{\mathbb {Z}}$$ contains two elements: $$g_2=5$$ with an angle $${\raise0.7ex\hbox{$1$} \!\mathord{\left/ {\vphantom {1 2}}\right.\kern-\nulldelimiterspace} \!\lower0.7ex\hbox{$2$}}\cdot \pi$$ and $$g_2^3=7$$ with angle $$\ {\raise0.7ex\hbox{$3$} \!\mathord{\left/ {\vphantom {3 2}}\right.\kern-\nulldelimiterspace} \!\lower0.7ex\hbox{$2$}} \cdot \pi$$. The coset $${\raise0.7ex\hbox{$1$} \!\mathord{\left/ {\vphantom {1 4}}\right.\kern-\nulldelimiterspace} \!\lower0.7ex\hbox{$4$}} +{\mathbb {Z}}$$ contains four elements: $$g_3=9$$ with an angle $${\raise0.7ex\hbox{$1$} \!\mathord{\left/ {\vphantom {1 4}}\right.\kern-\nulldelimiterspace} \!\lower0.7ex\hbox{$4$}} \cdot \pi$$ and $$g_3^3=11$$ with angle $${\raise0.7ex\hbox{$3$} \!\mathord{\left/ {\vphantom {3 4}}\right.\kern-\nulldelimiterspace} \!\lower0.7ex\hbox{$4$}} \cdot \pi$$ and $$g_3^5=13$$ with angle $${\raise0.7ex\hbox{$5$} \!\mathord{\left/ {\vphantom {5 4}}\right.\kern-\nulldelimiterspace} \!\lower0.7ex\hbox{$4$}} \cdot \pi$$ and $$g_3^7=15$$ with angle $${\raise0.7ex\hbox{$7$} \!\mathord{\left/ {\vphantom {7 4}}\right.\kern-\nulldelimiterspace} \!\lower0.7ex\hbox{$4$}} \cdot \pi$$. This principle continues with the coset $${\raise0.7ex\hbox{$1$} \!\mathord{\left/ {\vphantom {1 8}}\right.\kern-\nulldelimiterspace} \!\lower0.7ex\hbox{$8$}} +{\mathbb {Z}}$$ containing eight elements $$g_4=17$$ with an angle $${\raise0.7ex\hbox{$1$} \!\mathord{\left/ {\vphantom {1 8}}\right.\kern-\nulldelimiterspace} \!\lower0.7ex\hbox{$8$}} \cdot \pi$$, $$g_4^3=19$$ with an angle $${\raise0.7ex\hbox{$3$} \!\mathord{\left/ {\vphantom {3 8}}\right.\kern-\nulldelimiterspace} \!\lower0.7ex\hbox{$8$}} \cdot \pi$$ until $$g_4^{15}=31$$ with an angle $${\raise0.7ex\hbox{${15}$} \!\mathord{\left/ {\vphantom {{15} 8}}\right.\kern-\nulldelimiterspace} \!\lower0.7ex\hbox{$8$}} \cdot \pi$$. Generally we have:

1 $$\begin{aligned} g_n^k=2^n+k\qquad \text {with an angle (counterclockwise)}\qquad \pi \cdot {\raise0.7ex\hbox{$k$} \!\mathord{\left/ {\vphantom {k {2^{{n - 1}} }}}\right.\kern-\nulldelimiterspace} \!\lower0.7ex\hbox{${2^{{n - 1}} }$}} \end{aligned}$$

Now, “adding” two elements is achieved by the summation of their angles^9^. Let $g_m^{k_m}\le g_n^{k_n}$ be two elements. The sum of their angles $\alpha \left( g_m^{k_m}+g_n^{k_n}\right)$, which we note in short as $\alpha$, is given by:

$$\begin{aligned} \frac{\alpha }{\pi }=\frac{k_m}{2^{m-1}}+\frac{k_n}{2^{n-1}}=\frac{k_m\cdot 2^{n-m}+k_n}{2^{n-1}}=\frac{k_{m+n}}{2^{n-1}} \end{aligned}$$

It follows $m=\lfloor \log _2g_m^{k_m}\rfloor$, $n=\lfloor \log _2g_n^{k_n}\rfloor$, $k_m=g_m^{k_m}-2^{\lfloor \log _2g_m^{k_m}\rfloor }$, $k_n=g_n^{k_n}-2^{\lfloor \log _2g_n^{k_n}\rfloor }$. Three cases can be distinguished:

1. the angle $\alpha$ is an even integer, which means that $g_m^{k_m}+g_n^{k_n}=e=1$ is the identity element
2. the angle $\alpha$ is an odd integer, which means that $g_m^{k_m}+g_n^{k_n}=g_1=3$
3. the angle $$\alpha = {\raise0.7ex\hbox{${k_{{m + n}} }$} \!\mathord{\left/ {\vphantom {{k_{{m + n}} } {2^{{n - 1}} }}}\right.\kern-\nulldelimiterspace} \!\lower0.7ex\hbox{${2^{{n - 1}} }$}}$$ is a rational number (with a power of 2 as a denominator)

In the third case we need to reduce the fraction that represents the resulting angle $$\alpha$$. Moreover $$\alpha$$ must stay below $$2\pi$$ implying that the fraction $${\raise0.7ex\hbox{${k_{{m + n}} }$} \!\mathord{\left/ {\vphantom {{k_{{m + n}} } {2^{{n - 1}} }}}\right.\kern-\nulldelimiterspace} \!\lower0.7ex\hbox{${2^{{n - 1}} }$}}$$ stays below 2 and respectively the denominator $$k_{m+n}$$ cannot be equal or greater than $$2^{n}$$. For this reason we set $$k_{m+n}{:}{=}k_{m+n}\bmod 2^n$$. Reducing the fraction is achieved by determining the 2-adic valuation $$v_2(k_{m+n}\bmod 2^n)$$ of the denominator^15^, where $$2^{v_2(k_{m+n}\bmod 2^n)}$$ is the reduction factor:

$$\frac{\alpha }{\pi } = \frac{{{{k_{{m + n}} \,\bmod \,2^{n} } \mathord{\left/ {\vphantom {{k_{{m + n}} \,\bmod \,2^{n} } {2^{{v_{2} (k_{{m + n}} \,\bmod \,2^{n} )}} }}} \right. \kern-\nulldelimiterspace} {2^{{v_{2} (k_{{m + n}} \,\bmod \,2^{n} )}} }}}}{{{{2^{{n - 1}} } \mathord{\left/ {\vphantom {{2^{{n - 1}} } {2^{{v_{2} (k_{{m + n}} \,\bmod \,2^{n} )}} }}} \right. \kern-\nulldelimiterspace} {2^{{v_{2} (k_{{m + n}} \,\bmod \,2^{n} )}} }}}}$$

Using the basic relationship 1, we finally obtain the group sum of both elements $g_m^{k_m}$ and $g_n^{k_n}$ defining our Prüfer 2-Group operation:

2 $$\begin{aligned} g_m^{k_m}+g_n^{k_n}={\left\{ \begin{array}{ll} 1, &{} \text { if } {\raise0.7ex\hbox{$\alpha $} \!\mathord{\left/ {\vphantom {\alpha \pi }}\right.\kern-\nulldelimiterspace} \!\lower0.7ex\hbox{$\pi $}} \text { is an even integer }\\ 3, &{} \text { if } {\raise0.7ex\hbox{$\alpha $} \!\mathord{\left/ {\vphantom {\alpha \pi }}\right.\kern-\nulldelimiterspace} \!\lower0.7ex\hbox{$\pi $}} \text { is an odd integer}\\ { \frac{2^{n}+k_{m+n}\bmod 2^n}{2^{v_2\left( k_{m+n}\bmod 2^n\right) }}}, &{} \text {otherwise} \end{array}\right. } \end{aligned}$$

Recall that $n=\lfloor \log _2g_n^{k_n}\rfloor$ and $k_{m+n}=k_m\cdot 2^{n-m}+k_n$. Thus the equation for $k_{m+n}$ that only depend on both group elements $g_m^{k_m}$ and $g_n^{k_n}$ becomes $k_{m+n}=g_m^{k_m}\cdot 2^{\lfloor \log _2g_n^{k_n}\rfloor -\lfloor \log _2g_m^{k_m}\rfloor }+g_n^{k_n}-2^{\lfloor \log _2g_n^{k_n}\rfloor +1}$

**Figure 3:**
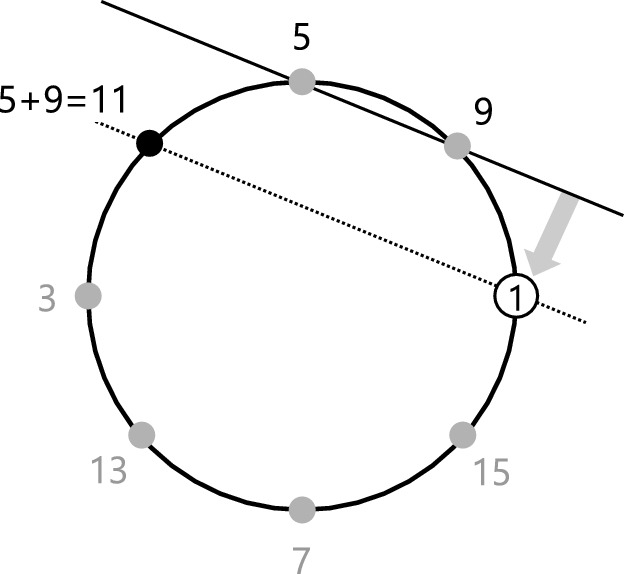
Geometric group law on the elements of our labeled Prüfer 2-Group.

Figure 3 displays the acting principle of our Prüfer 2-Group operation in a geometric way. This principle is based on the *Group Law on Conics*, which has been introduced by Franz Lemmermeyer^16,17^: We fix the vertex labeled with 1 (the identity element *e*) and define the sum of two elements $g_i$ and $g_j$ on the unit circle as the second vertex of intersection of the circle with the parallel to $g_ig_j$ through *e*. In the example from Figure 3 we have $i=2$ and $j=3$ and the sum of $g_2$ and $g_3$ is $g_3^3=11$ (5+9=11). Table 1 shows for the first 19 elements of our Prüfer 2-Group their summation. The group operation $+$ is implemented in Listing 1. Accordingly, add [5,9]=11 for example, and add [11,15]=5, add [13,19]=29 and so on.

**Listing 1:**
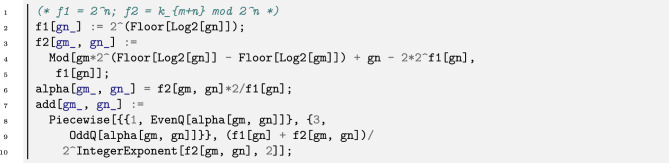
Listing for the operator of our Prüfer 2-Group as per equation 2

Table 1 shows the result obtained from the “addition” of two elements up to 19, *e.g.* $13+7=11=5+9$, where we can replace 9 with $7+11$ which leads to $13+7=5+7+11$ and therefore $13=5+11$.

**Table 1:** Addition Table arranging the sums of the first 19 group elements.

$+$	1	3	5	7	9	11	13	15	17	19	$\ldots$
1	1	3	5	7	9	11	13	15	17	19	
3		1	7	5	13	15	9	11	25	27	
5			3	1	11	13	15	9	21	23	
7				3	15	9	11	13	29	31	
9					5	3	7	1	19	21	
11						7	1	5	23	25	
13							5	3	27	29	
15								7	31	17	
17									9	5	
19										11	
$\vdots$											$\ddots$

Now that the coordinate system is well defined, the final step of diophantine imaging consists of mapping the known solutions to important diophantine equations onto the labeled subgroup of the Unit circle, which allows us to use referentials in any base, be it whole (*e.g.* 2) - in which case, if the number is prime, the referential will be a Prüfer group - or real (*e.g.* $\pi$).

For an integer *d*, here we will compare the plots of integer triplets $\{a, b, c\}$ solving either $a^3+b^3+c^3=d^3$ (cubic quadruples) or $a^3+b^3+c^3=d$ (sums of three cubes), in the bases 2, 3; 7, $\pi$, $\phi$ and *e*. To further refine the plots, we will distinguish the so obtained triangles by the range of their surface as divided by that of the circle itself (in $\%$ and leaving the impossible $42\%+$ range for control). The complete Mathematica Notebooks and used open datasets are available at GitHub and Kaggle^18,19^ (see Section “Data Availability”).

## Data retrieval and merge

**Figure 4:**
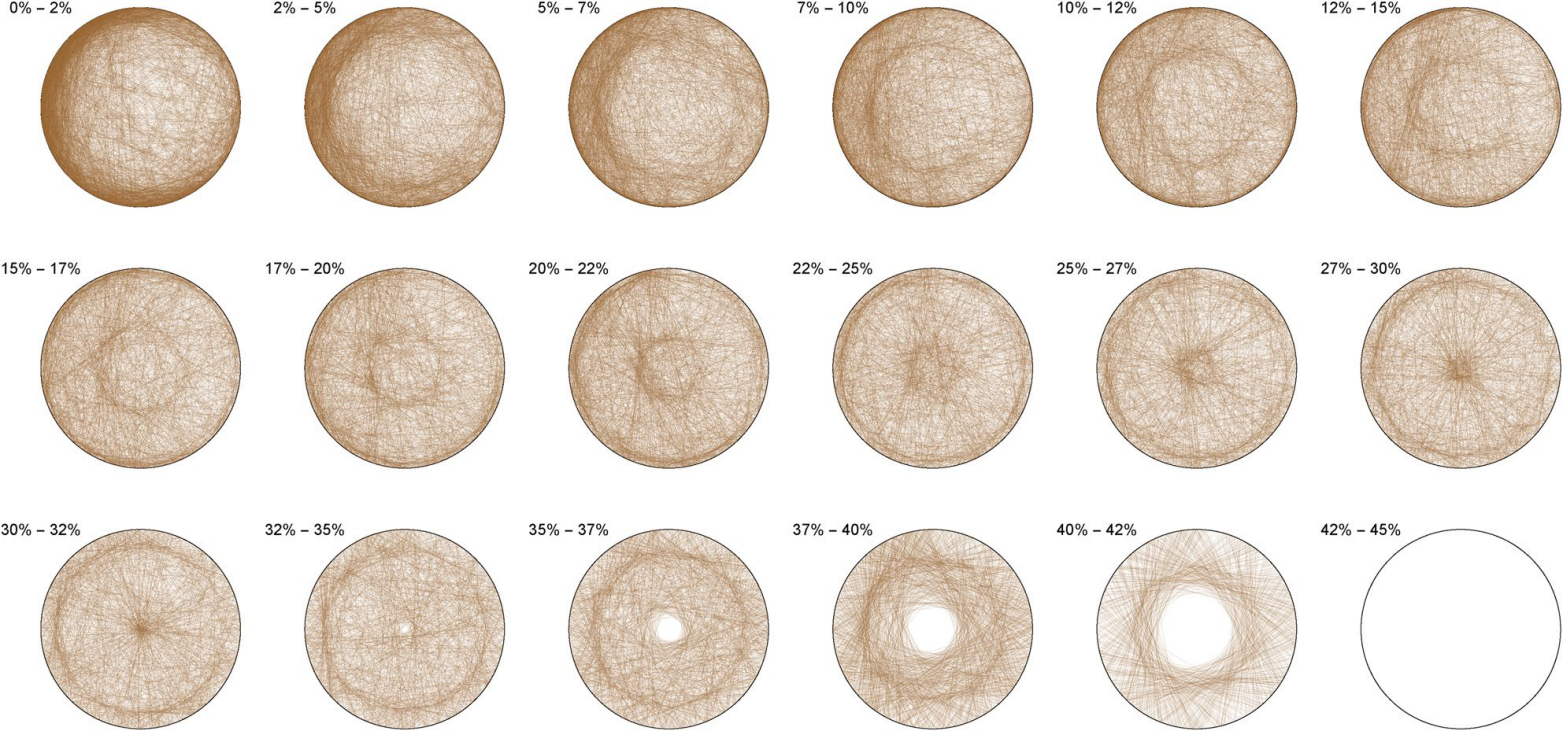
Cubic quadruples in base 2.

**Figure 5:**
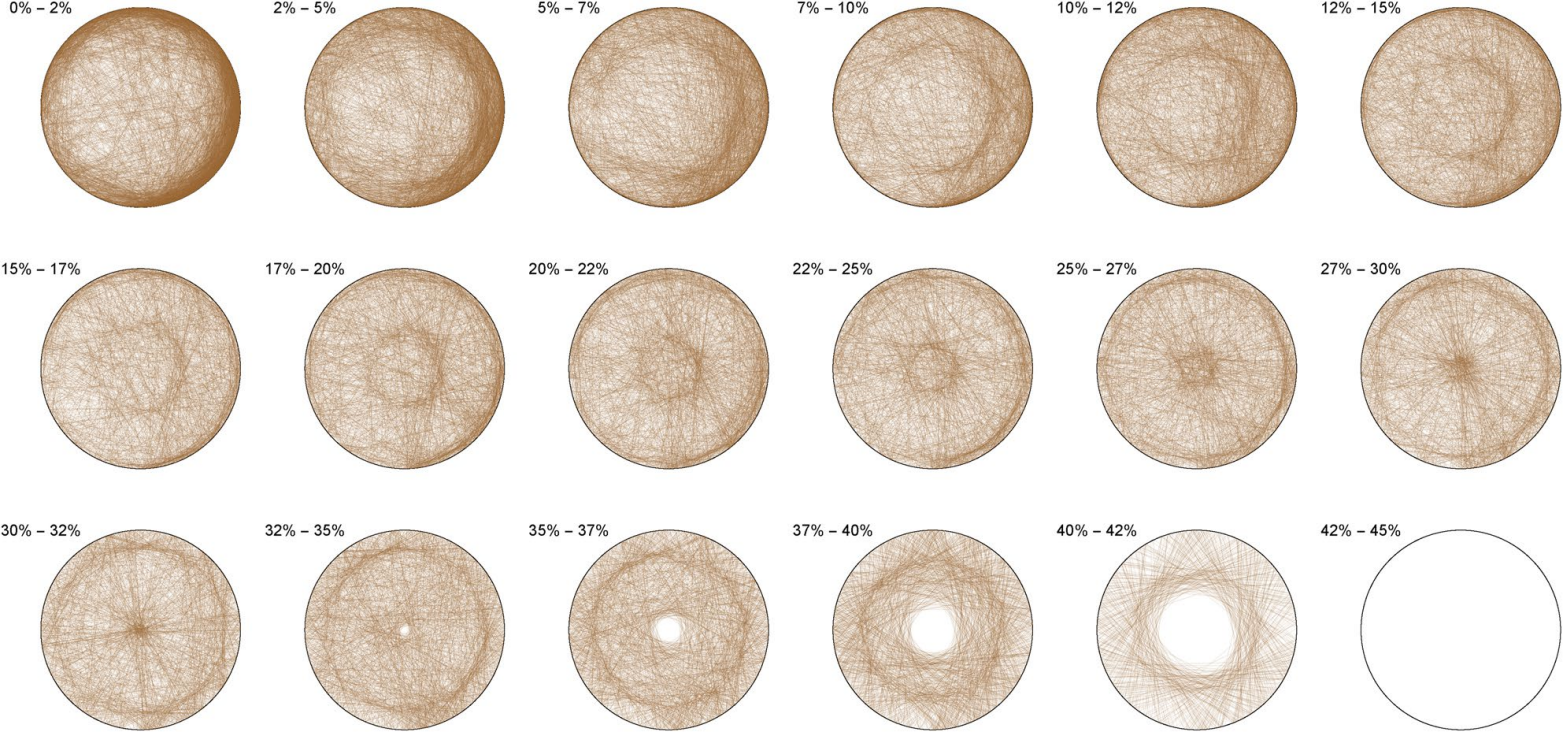
Cubic quadruples in base 3.

**Figure 6:**
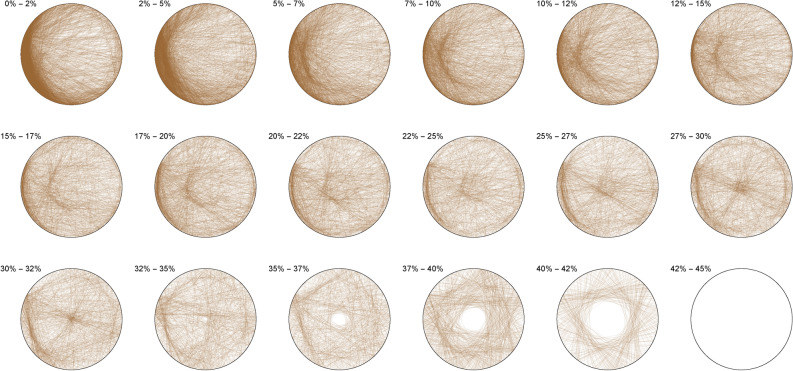
Cubic quadruples in base 7.

**Figure 7:**
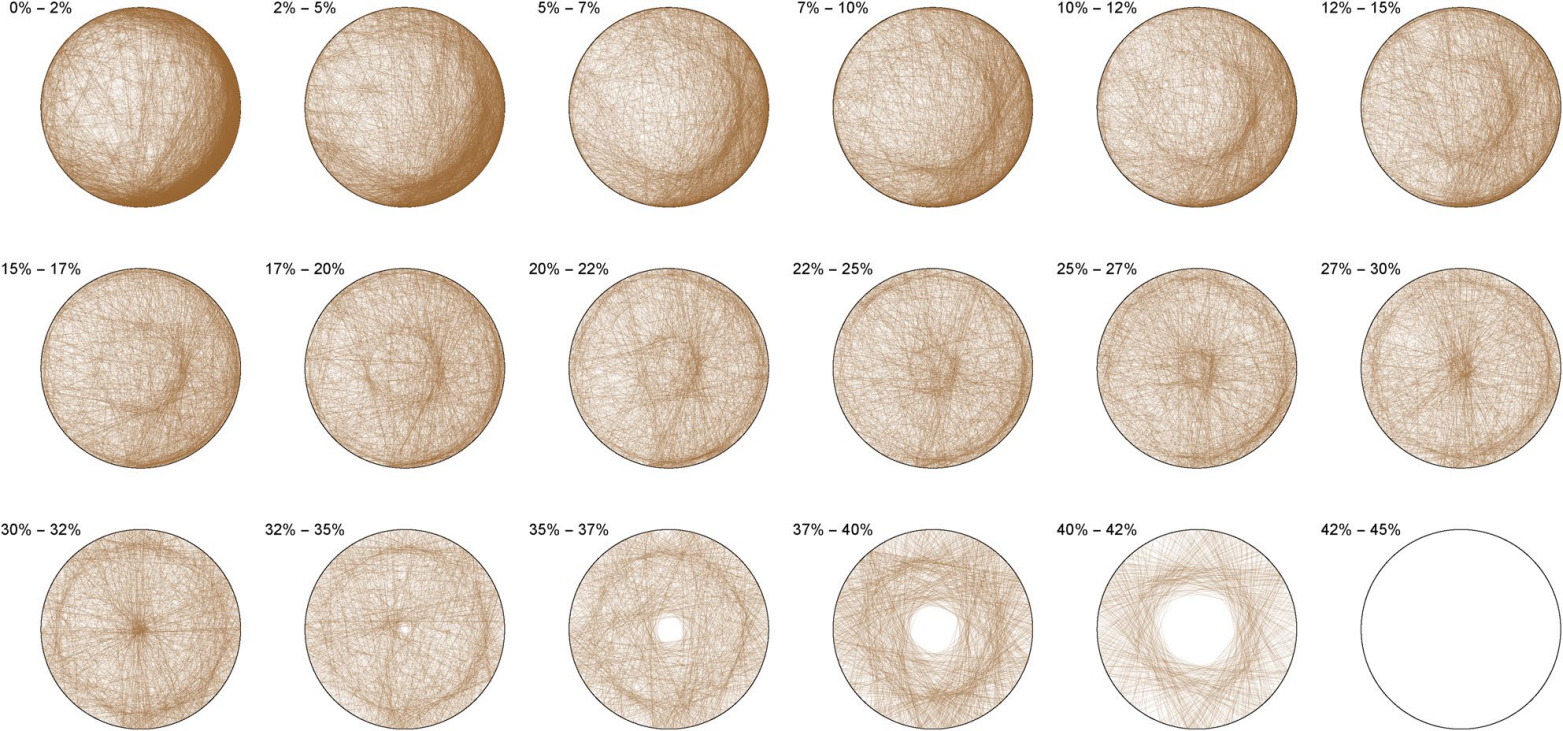
Cubic quadruples in base $\pi$.

**Figure 8:**
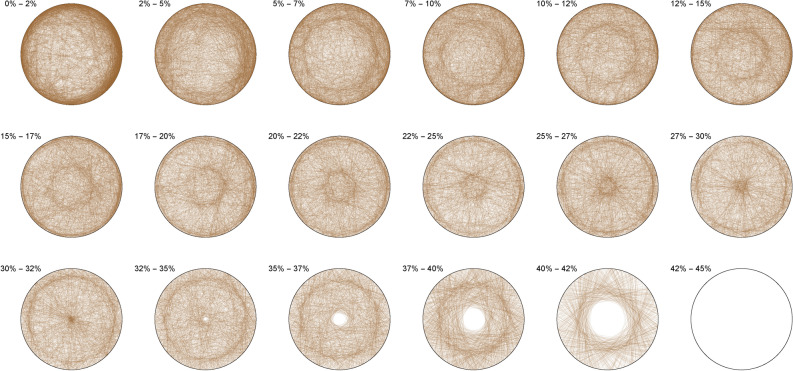
Cubic quadruples in base $\phi$.

**Figure 9:**
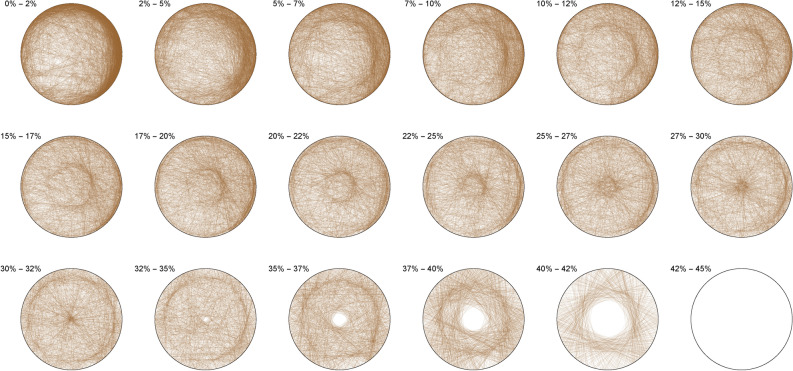
Cubic quadruples in base *e*.

Figures 4, 5, 6, 7, 8, 9 are based on the dataset^19^ which is a merge of the files provided by Jaroslaw Wroblewski^20^ containing a list of known solutions for Equal Sums of Powers (3, 1, 3) below 1, 000, 000. Wroblewski’s (merged) data set is publicly available at Kaggle. The script for downloading and merging Wroblewski’s files is available at GitHub^18^, see Listing 2.

**Figure 10:**
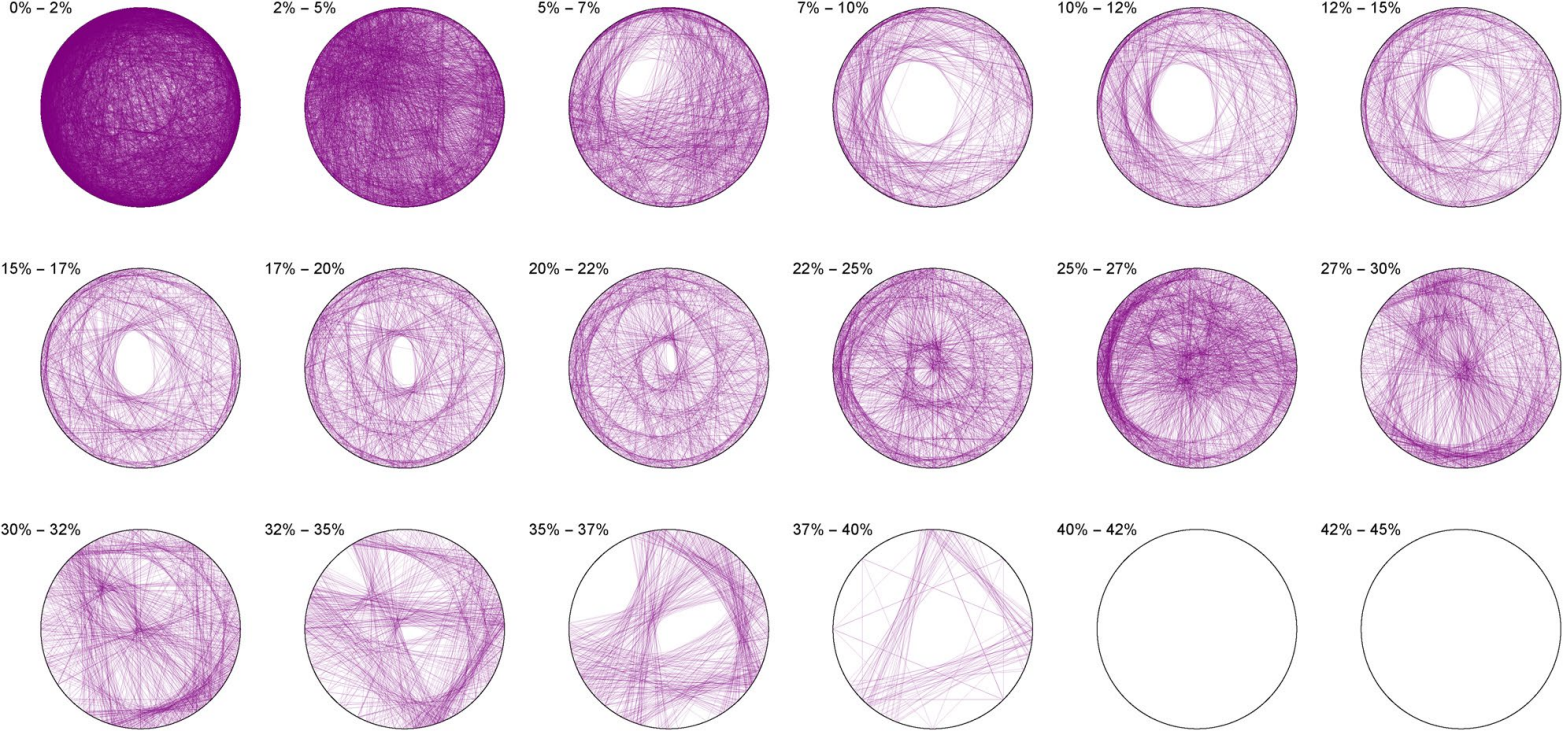
Integer cubes in base 2.

**Figure 11:**
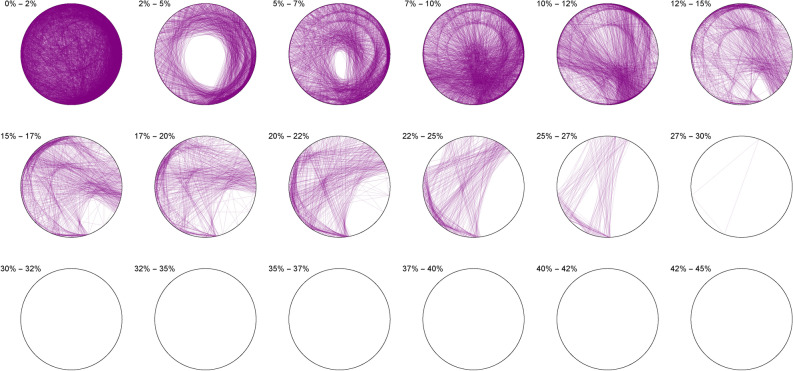
Integer cubes in base 3.

**Figure 12:**
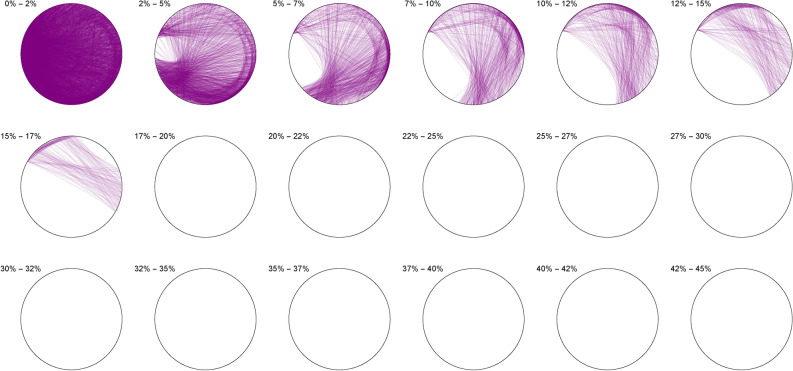
Integer cubes in base 7.

**Figure 13:**
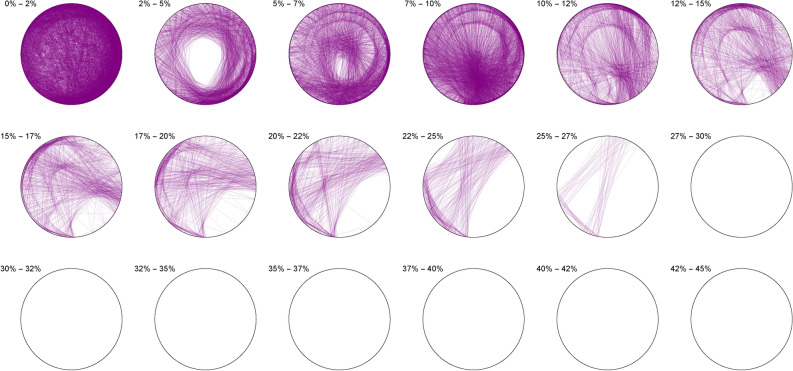
Integer cubes in base $\pi$.

**Figure 14:**
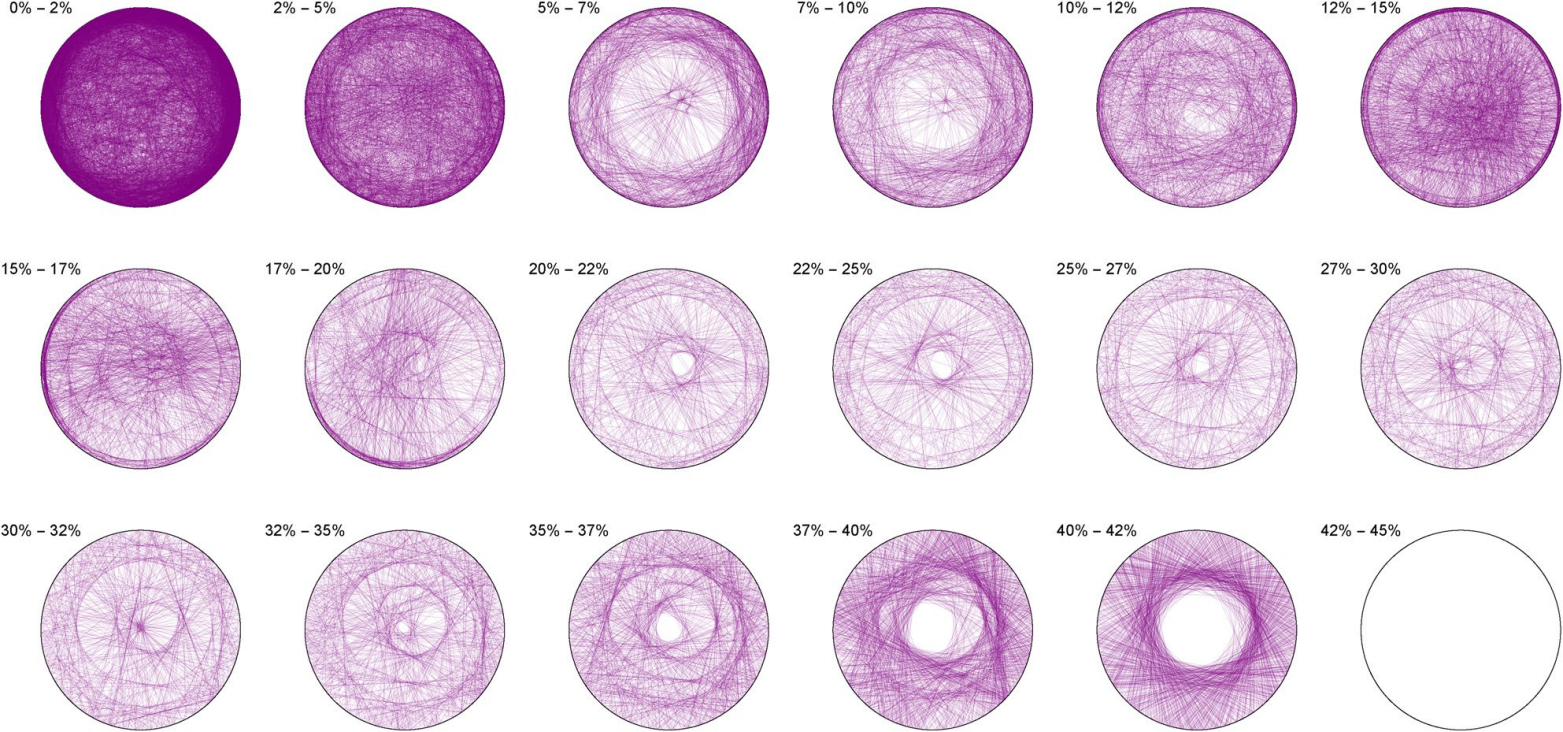
Integer cubes in base $\phi$.

**Figure 15:**
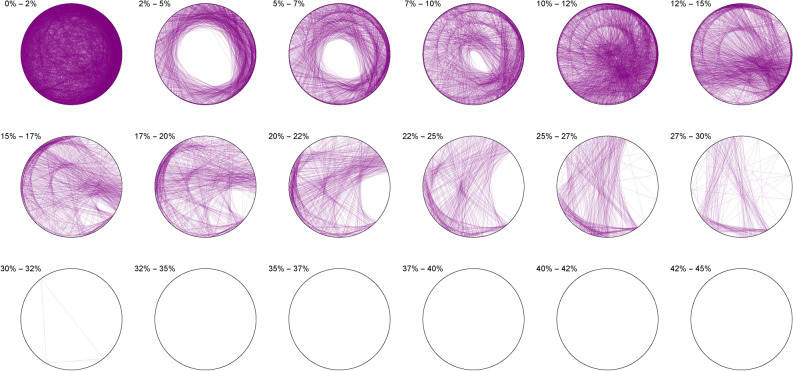
Integer cubes in base *e*.

Figures 10, 11, 12, 13, 14, 15 are based on the dataset listing solutions to sums of integer cubes for $n<1000$ neither a cube nor twice a cube (search bound: $10^{14}$) provided by Andreas-Stephan Elsenhans and Joerg Jahnel^21^. The dataset of Elsenhans and Jahnel was formerly available at University of Göttingen. A copy is available at GitHub.

**Listing 2:**
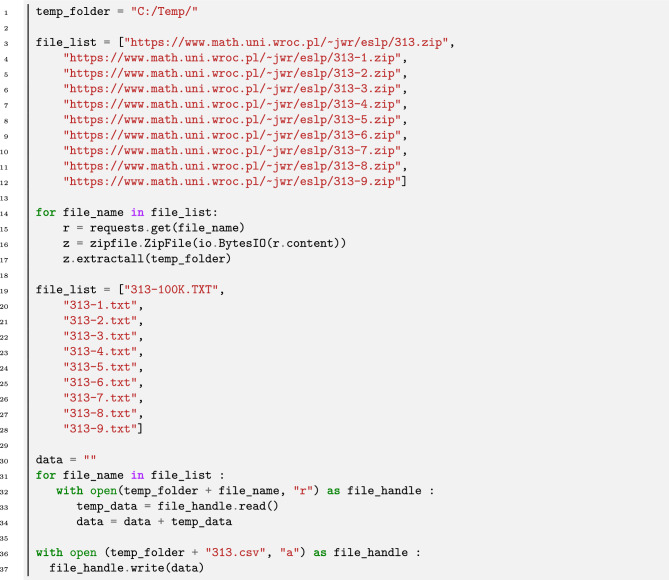
Wroblewski’s Equal Sums of Powers (3,1,3) data retrieval and merge

## Results

The following pictures visually illustrate the solutions of the Diophantine equations. We display a percentage range above each circle, which indicates the percentage coverage of the circle area by each triangle. For example, 0% – 2% means that in this particular circle only triangles are drawn, each of which covers not more than 2% of the circle area.

The Diophantine imaging of cubic quadruples in base 2 (Figure 4) reveals a seemingly ergodic behavior.

Figure 5 shows the same ergodic behavior in base 3

While Figure 6 already outlines a slight asymmetry. Imaging in non-integer bases like $\pi$ (Figure 7), $\phi$ (Figure 8) or *e* (Figure 9) does not reveal any obvious change in distribution: the all three bases will mostly preserve the ergodicity, which we discover is a comparative property of cubic quadruples as opposed to sums of three cubes.

We thus observe a seemingly ergodic distribution of the solutions of cubic quadruples in all the studied bases, prime (2; 3; 7), algebraic ($\phi$) or transcendental ($\pi ; e$) albeit with a noticeable asymmetry for narrow triangles (20% and below) in base 7 where certain prefix classes appear more favored. However, such general ergodicity is not at all occurring for the sums of integer cubes, in any base.

The Diophantine imaging of sums of integer cubes in base 2 (Figure 10) reveals a very precise fingerprint with quasi-envelopes emerging even for narrow triangles, and such a distribution is very different from that in base 3 (Figure 11)

Base 7 (Figure 12) is in itself very asymmetric and immediately distinguishable from base 3 and this uniqueness pursues in the chosen non-integer bases like $\pi$ (Figure 13)

Very interestingly, imaging in base $\phi$ (Figure 14) output the most ergodic distribution of the solutions of sums of three cubes, which a comparison with base *e* (Figure 15) will make all the more evident. As of yet, we have absolutely no explanation as to why base $\phi$ is so particular.

## Discussion and conclusion

### A proof of concept for diophantine Imaging

The purpose of this article was to communicate a general method for plotting known solutions of diophantine equations in a manner that would well-behave arbitrarily large numbers and offer new insight into their ergodicity or lack thereof. In this sense, we considered the comparison of the distribution of cubic quadruples and sums of three integer cubes would constitute a clear yet original proof of concept of Diophantine Imaging, by offering novel yet both easily reproduced and easily grasped observations of the arithmetic geometry of this class of equations.

The first of such observations is the seeming base-invariant ergodicity of the distribution of the triplets solving a cubic quadruple, and the comparative negentropy of the triplets solving a sum of integer cube. Given a certain base and class of surface, entire families of prefixes seems to be forbidden in the distribution of solutions of integer three cubes, while this appears not to be the case at all of cubic quadruples. Moreover, given a certain base, entire classes of surface areas also seem to be banned for the sums of three cubes, while they are not for cubic quadruples. More interestingly, we found that there were certain bases ( $\phi$ here) that could better-behave the negentropic distribution for the sums of three cubes, calling for further investigation and more refined methods of imaging.

### Informing original conjectures and programs

As a practical tool for guiding the scientific attention of the working mathematician and computational number theorist, the purpose of Diophantine Imaging may be really fulfilled when it has informed sufficiently clear testable conjectures or even research programs. From the data we assembled and visualised, we may already - yet still loosely - outline a few of them here:

***A loose conjectural roadmap towards Diophantine Galois Theory***

(i) Are the solutions of cubic quadruples organised as conference graphs?
(ii) Can we formulate a Diophantine Ramsey theory identifying necessary prefix conditions for sums of integer cubes?
(iii) Furthermore, are there explicit formulas (like the Euler characteristic) fixing topological invariants based on the prefixes of the solutions of sums of integer cubes?
(iv) Can the synthesis of the former Ramsey-theoretical and latter Geometric approach inform the definition of an *ad hoc* Galois theory for these classes of diophantine problems?
(v) Are there explicit three-dimensional parametric envelopes defining the distribution of each classes of surface area?
(vi) Is there a perfect base maximising ergodicity for the solutions of sums of three cubes ? Could this base be algebraic or transcendental? Would such a base exist for other difficult classes of diophantine problems?

In particular the latter search for “bases fixing (or “most fixing”) the ergodicity of” such and such distribution of diophantine solutions would appear as an epistemological kin of the already well-established search for measure-preserving transformations in ergodic theory; it was indeed the initial intent of Diophantine Imaging to modestly contribute to the “remarkable effectiveness of ergodic theory in number theory”.

It has been known since Matyasevitch (1970) and in particular the consolidated MRDP theorem^22^ that no general method for establishing the solvability of a diophantine equation with rational coefficients exist. From an epistemological perspective, we see this strong result as an incentive to further refine the distinction between classes of diophantine equations, and to break ground for local *ad hoc* theories specifically adapted to certain classes. We started from the case of sums of three integer cubes because they are both considered extremely difficult, yet at the edge of the state of the art and still offer very large datasets of known solutions. From the data structuring we laid out in this article, we can but only recommend a two-pronged theoretical roadmap,one starting with Graph Theory with the intent of reaching groundbreaking results strong enough to establish a dedicated Ramsey Theory based on prefixes, and the other starting from the search of topological invariants and characteristics, with the intent to merge the two to finally break the ground for a dedicated Diophantine Galois Theory.

### Technical perspectives

The reason we called the technique introduced in this article a form of “imaging” is that we wanted to attract the reader’s attention on its practical improvability; techniques and technologies may call for more regular updates and upgrades than mere general methods. As we wanted, in the previous subsection, to lay down an indicative theoretical roadmap towards Diophantine Galois theory, we would like to conclude here on the more practical and immediate improvements we believe should be made to Diophantine Imaging in future studies and proofs of concept.

***A technical roadmap for diophantine imaging***

(i) imaging triplets solving $a^4+b^4+c^4=d^4$
(ii) 3D imaging to further refine complex envelopes into parametric spirals (where the z-axis is the number of digits in the represented number)
(iii) multi-parameter imaging beyond just bases and classes of surface areas
(iv) automatic parameter suggestion and clustering based on artificial intelligence and in particular evolutionary algorithms
(v) automatic search for bases fixing the ergodicity of certain distributions by artificial intelligence, but this time using machine learning

## Data Availability

The datasets analysed during the current study are available in the Kaggle repository, https://www.kaggle.com/datasets/eldarsultanow/equal-sums-of-powers-313 and in the GitHub repository, https://github.com/Sultanow/pedal_curves/tree/main/mathematica/datasets.

## References

[1] Rassokhin, D. Diophantine-based encryption.

[2] Okumura, S. A public key cryptosystem based on diophantine equations of degree increasing type (2015).

[3] Engcot, M. K. C. Cryptography using linear diophantine equation (2015).

[4] Booker A (2019). Cracking the problem with.

[5] Matić L (2012). Non-existence of certain diophantine quadruples in rings of integers of pure cubic fields. Proc. Jpn. Acad. Ser. A Math. Sci..

[6] Hürlimann W (2017). Permutation invariant properties of primitive cubic quadruples. Ramanujan J..

[7] Matheus C (2009). The remarkable effectiveness of ergodic theory in number theory. Ensaios Math..

[8] Rahn A, Sultanow E, Henkel M, Ghosh S, Aberkane I (2021). An algorithm for linearizing the collatz convergence. Mathematics.

[9] Baez, J. Prüfer 2-group (2014).

[10] Schmidt R (1994). Subgroup Lattices of Groups.

[11] Friedman, H. M. Metamathematics of ulm theory. Tech. Rep. S1574-0358(04)70754-6, Ohio State University (2001).

[12] Comfort, W. W., Hofmann, K.-H. & Remus, D. Recent progress in general topology. In Hušek, M. & van Mill, J. (eds.) *Recent Topological Developments on Topological Groups* (Elsevier Science, 1992).

[13] Grove, L. C. *Algebra* (Academic Press, 1983).

[14] Gilbert, W. J. & Nicholson, W. K. *Modern Algebra with Applications* (John Wiley & Sons, 2003), 2 edn.

[15] Herwig TC (2011). The p-adic Completion of q and Hensel’s Lemma.

[16] Lemmermeyer, F. Conics – a poor man’s elliptic curves. https://arxiv.org/abs/math/0311306 (2003).

[17] Lemmermeyer, F. Pell Conics: An Alternative Approach to Elementary Number Theory. https://www.mathi.uni-heidelberg.de/~flemmermeyer/pell/bfc02.pdf (2012).

[18] Sultanow, E. pedal_curves (2022).

[19] Sultanow, E. Equal sums of powers (3,1,3) (2022).

[20] Wroblewski, J. Equal sums of powers - tables (2008).

[21] Elsenhans, A.-S. & Jahnel, J. List of solutions of x$$^3$$ + y$$^3$$ + z$$^3$$ = n for n $$<$$ 1000 neither a cube nor twice a cube.

[22] Davis, M., Matijasevic, Y. & Robinson, J. Diophantine equations: Positive aspects of a negative solution. In *Mathematical Developments Arising From Hilbert Problems* (American Mathematical Society, American Mathematical Association, 1976).

